# Learning of bimanual motor sequences in normal aging

**DOI:** 10.3389/fnagi.2015.00076

**Published:** 2015-05-08

**Authors:** Rashmi Bhakuni, Pratik K. Mutha

**Affiliations:** ^1^Department of Biological Engineering, Indian Institute of Technology GandhinagarAhmedabad, Gujarat, India; ^2^Centre for Cognitive Science, Indian Institute of Technology GandhinagarAhmedabad, Gujarat, India

**Keywords:** motor learning, serial reaction time task, bimanual actions, aging, skill

## Abstract

While it is well accepted that motor performance declines with age, the ability to learn simple procedural motor tasks appears to remain intact to some extent in normal aging. Here we examined the impact of aging on the acquisition of a simple sequence of bimanual actions. We further asked whether such learning results from an overall decrease in response time or is also associated with improved coordination between the hands. Healthy young and old individuals performed a bimanual version of the classic serial reaction time task. We found no learning deficit in older adults and noted that older subjects were able to learn as much as young participants. We also observed that learning in both groups was associated with an overall decrease in response time, but switch cost, the increase in response time when a switch in hands was required during sequence execution, did not decrease with learning. Surprisingly however, overall switch cost was lower in the older group compared to the younger subjects. These findings are discussed in the context of interactions between procedural and declarative memory, reduced interhemispheric inhibition and more symmetric cortical activation during motor performance in normal aging.

## Introduction

A large body of research has been dedicated to understanding the impact of normal aging on sensorimotor function. These studies have generally shown that motor performance declines with age (Grabiner and Enoka, 1995; Cole et al., 1999; Krampe, 2002); this occurs due to a variety of factors that range from loss of muscle strength (Evans and Campbell, 1993; Evans, 2010) to changes in brain structure and volume (Haug and Eggers, 1991; Walhovd et al., 2005). Concomitant with this decrement in motor performance, several studies have also demonstrated age-related decline in cognitive function (Rypma et al., 2001; Pettigrew and Martin, 2014). In particular, formation of memories associated with facts and events, often referred to as declarative memory, has been consistently shown to be impaired in older individuals (Grady et al., 1995; Albert, 1997; Fjell and Walhovd, 2010). Curiously however, a number of studies have shown that the capacity to develop motor memories, or to improve motor performance with practice, does not decline with age (Buch et al., 2003; Smith et al., 2005; Brown et al., 2009; Anguera et al., 2011; Wang et al., 2011). For example, Wang et al. (2011) recently showed that older adults were able to learn and modify their motor output to account for the effects of a novel mapping between hand motion and its visual feedback to the same degree as young individuals. Such findings have been reported in several other studies as well. In fact, Heuer and Hegele (2008) suggested that any observed differences between young and older adults in such motor learning tasks could be explained by differences in cognitive factors rather than the capacity for learning itself.

In contrast to tasks that require learning to adapt motor output in response to novel movement conditions, findings about how aging impacts the learning of skilled sequential actions, as might be required for tasks like typing or playing the clarinet, are mixed. While some studies have suggested that learning of movement sequences appears to be intact in normal aging (Howard and Howard, 1992; Daselaar et al., 2003; Shea et al., 2006; Spencer et al., 2007; Brown et al., 2009; Romano et al., 2010), others have identified specific age-related deficits in the ability to learn sequential movements (Curran, 1997; Willingham and Goedert-Eschmann, 1999; Feeney et al., 2002; Willingham et al., 2002; Howard et al., 2004). For example, in a unimanual sequence learning paradigm (serial reaction time task, SRT), Brown et al. (2009) showed intact sequential skill acquisition in older adults, and in fact reported that the magnitude of their acquired skill was greater than younger participants. While such enhanced skill learning relative to young individuals has not been demonstrated in a bimanual context, some studies have demonstrated that older adults are capable of learning bimanual sequential actions at least as well as young adults. For instance, Howard and Howard (1997) examined the ability to learn a sequence of finger movements associated with visually presented stimuli and demonstrated a comparable reduction in response time in young and old individuals as a consequence of learning. Further, a similar decrement in terms of response times was noted across the groups when a series of random finger movements had to be performed after learning. In contrast however, some other studies have noted sequence learning deficits in the elderly, particularly when task or sequence complexity is increased (Feeney et al., 2002; Howard et al., 2004), or when explicit knowledge about the sequence is provided (Willingham and Goedert-Eschmann, 1999; Willingham et al., 2002). It has been suggested that age-related cognitive decline may contribute to such deficits (Salthouse, 1996; Bo et al., 2009) and that these deficits can be mapped to an age-associated deterioration in corticostriatal networks (Rieckmann and Bäckman, 2009; Rieckmann et al., 2010). However, given the diversity of task conditions across studies, it is difficult to paint a coherent picture of the exact impact of aging on the ability to learn bimanual sequential skills.

Another important question with regard to bimanual motor learning is whether learning is associated with improved performance of individual limbs, or also improved coordination between limbs. This question, surprisingly, has not been investigated in detail. Further, if indeed bimanual coordination does improve with learning, whether and how aging impacts this improvement in coordination remains unknown. Note that there has been a large body of research investigating the impact of aging on coordination itself (Stelmach et al., 1988; Swinnen, 1998; Serrien et al., 2000; Bangert et al., 2010; Seidler et al., 2010). These studies have shown that bimanual coordination becomes poorer with age, particularly in conditions where significant executive control is required. In this context, investigating whether coordination is improved with training is an important question from a translational standpoint when attempting to improve motor performance in both healthy as well as neurologically impaired elderly individuals.

Our aim here was to begin investigating these issues. We first examined the impact of aging on the acquisition of a sequence of bimanual actions. We then asked whether learning is associated with improved coordination between the limbs. Finally, we enquired whether aging would affect this improvement in coordination. We required healthy young and old adults to perform a SRT task in which sequence execution required responses with the index and middle fingers of each arm, under conditions in which no explicit knowledge about the presence of a sequence was provided. Based on prior work, we expected sequence learning under these simple task conditions to be intact in older adults. To assess changes in coordination with learning, we studied whether the time cost associated with switching between hands when performing bimanual actions would change as a consequence of learning. We also examined whether the pattern of change would be different between the young and the older groups.

## Materials and Methods

### Ethics Statement

The study was approved by the Institutional Ethics Committee of the Indian Institute of Technology Gandhinagar and subjects provided informed consent for participation.

### Participants

Fifteen young healthy adults (mean age 22.73 years, 4 females and 11 males) and fifteen healthy elderly (greater than 60 years of age) individuals (mean age 63.73 years, 2 females and 13 males) participated in the experiment. Subjects reported no peripheral movement restrictions or orthopedic injuries and were excluded if they had any history of neurological problems. All subjects were self-reported right-handers and had normal or corrected to normal vision. Young participants were primarily college undergraduates; older adults were recruited from in and around the institute. One young and three older subjects had large error rates (greater than mean + 2 SD) and were therefore excluded from the analysis. Note however that since the learning results were based only on the correct trials (see below), the overall trends in the results on remained the same if these subjects were included. However, since error rate itself was a critical measure, we decided to exclude these subjects from all analyses.

### Experimental Setup and Task

**Figure 1:**
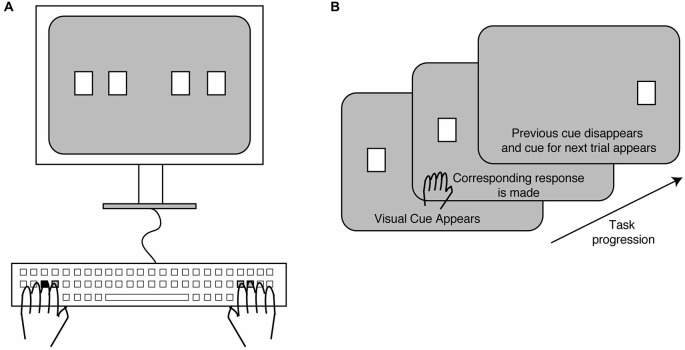
**Experimental setup and task. (A)** Subjects sat facing a computer screen on which stimuli (white squares) were displayed in one of four locations. These locations corresponded to specific keys on the keyboard (shown in gray), which were to be pressed with the index and middle fingers of the left and right hands. The remaining keys were removed from the keyboard. **(B)** A trial began with one of the stimuli appearing on the screen. The corresponding key was pressed upon which the stimulus disappeared and the next stimulus was displayed in a new location.

All participants performed a bimanual variant of the SRT task. Participants sat in a dark room facing a computer monitor placed at a distance of about 60 cm from their face and rested both their hands on a tabletop (Figure 1A). A keyboard was used as a response pad. Participants observed white square stimuli appear one at a time on the computer screen in one of four different horizontal positions. Each stimulus location corresponded to either the S, D, J or K keys on the keyboard. Subjects used the middle and index fingers of the left hand when pressing the S and D keys respectively, while they used the index and middle fingers of their right hand when pressing the J and K keys respectively. Thus, the four stimulus locations were mapped to four fingers, two of each hand. A displayed stimulus square was set to disappear as soon as subjects made the response and a new stimulus was displayed immediately afterwards (Figure 1B). If subjects failed to respond within 3 s of stimulus presentation, the displayed stimulus disappeared and the next stimulus was shown.

The task comprised of an initial familiarization block of 24 trials, where a trial is defined as a single key press. These pseudorandom trials were simply to acquaint subjects to the setup and nature of the stimuli and responses they needed to make. During this set of trials, the stimuli occurred in no particular order with the only constraint being that all four fingers had to be used an equal number of times. Following the familiarization block, the stimuli were presented in a sequential or a pseudorandom manner. Sequential presentation comprised of a 12-element sequence such that each finger being used during the task was used thrice. Subjects were not informed that stimuli would be presented sequentially. We used the following sequence: LI—LM—RM—RI—RM—LM—LI—RI—RM—RI—LI—LM where L and R correspond to the left and right hand, and I and M correspond to the index and middle fingers respectively. As can be seen from the sequence elements, execution of this sequence required use of both hands and also a switch between hands. Prior to, and after 40 repetitions of the 12-element sequence (480 trials), subjects performed a set of 12 trials in which the stimuli were presented in a pseudorandom order. Finally, subjects performed two more sets of the 12-element sequence after the second presentation of the random trials. Response times were recorded for each trial.

### Data Analysis

Our primary dependent measure was response time. To examine learning of the sequence, we assessed the change in response time as function of sequence number as well as the difference between response times of the random and sequence trials. A single mean response time was then calculated per sequence by averaging the response times for the correct trials of each sequence (1.45% of all responses were errors). This sequence specific response time was averaged across all participants from each group to yield a group specific response time for that sequence. The effect of outliers was reduced by removing trials that were identified using Tukey’s outlier box plots. In this analysis, an interquartile range (IQR) was first calculated and data points below Q1 − 1.5 * IQR and above Q3 + 1.5 * IQR were noted as outliers (Q1 and Q3 correspond to the first and third quartile while IQR corresponds to the inter-quartile range). In our data, approximately 3% of all trials were identified as outliers using this method.

In order to examine the change in response time associated with switching of hands during execution of the sequence, we performed an analysis similar to that of Trapp et al. (2012). We first created 8 time bins of 5 sequences each (40 sequence presentations total). The bin-wise analysis enabled us to examine the changes in the switch cost over chunks of sequences rather than at the individual sequence level. This provided a gross estimate of how, if at all, switch cost changed over the course of learning. We averaged the response times separately for each of the 12 key presses across all the 5 sequences included in a bin, and also across all participants in a group. For example, we averaged the response times for the first left index finger press across the 5 sequences in the first bin, across all participants. This was repeated for all the other key presses in the training sequence, for all bins. For each bin, we then considered sequential responses in which there was a hand switch (left to right or right to left) and calculated the change in response time associated with making that switch as 100 − (mean response time before switch/mean response time after switch) * 100. We expected this “switch cost” to increase with a change in hand, and our interest was in examining whether it would decrease with learning. In other words we investigated whether, as a consequence of learning, participants would learn to switch faster between hands, and whether there would be a difference between the young and older groups. We also considered an alternate analysis of switch cost where instead of just the trial preceding the switch, we used all trials that did not require a switch between hands. Switch cost was then calculated as described above, but the mean response time for all the no-switch trials was used.

To compare the progress of sequence learning across groups statistically, we first performed a two-way ANOVA with group (young, old) and sequence number (all 40) as factors. Further, to investigate group differences in the magnitude of learning, we compared the change in response time from the first sequence to the last using another two-way group (young, old) × sequence (first, last) ANOVA. We also compared the groups on a measure of “skill” defined as the difference between the response times of the second random trial presentation and the last sequence trial (S40) (Brown et al., 2009). The potential change in hand switch cost with learning was investigated using a group (young, old) × bin (1 through 8) ANOVA. Tukey’s *post hoc* tests were conducted when warranted by significant main effects.

## Results

### Overall Change in Response Time

**Figure 2:**
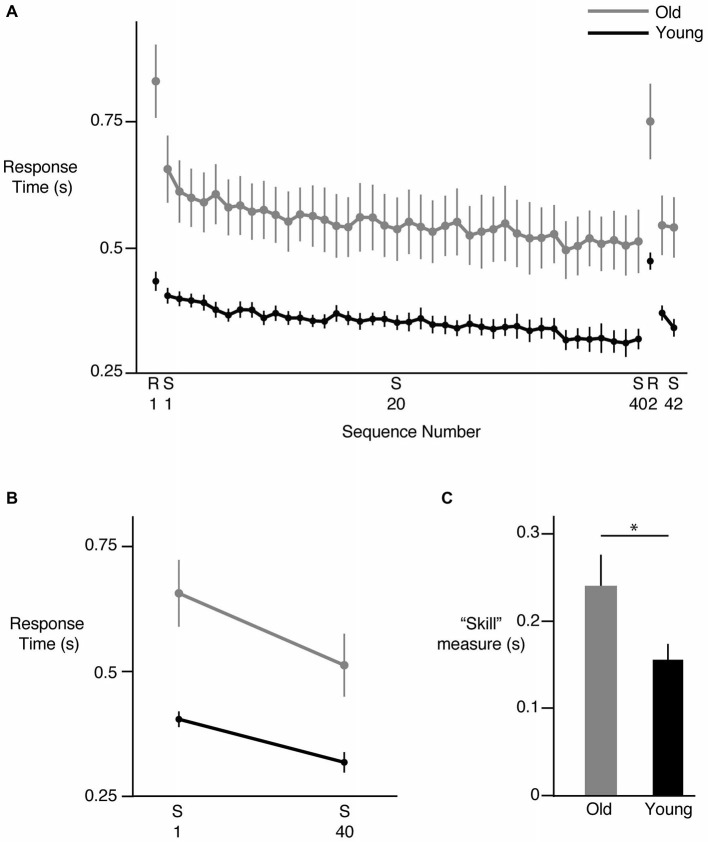
**Sequence specific learning. (A)** Reduction in response time across sequences. Each point represents the mean response time for all 12 key presses in a sequence, across all subjects. S represents the training sequence, while R represents a random sequence of key presses. Young subjects are shown in black, while older subjects are shown in gray. In both groups, response times decreased from S1 to S40, then increased on presentation of R2, and then decreased again on S41 and S42, indicating development of sequence specific learning. **(B)** Net change in response time from S1 to S40 in the two groups. The magnitude of change was greater in the old group. **(C)** Difference in sequence specific “skill” learning between the old (gray) and young (black) groups. All data shown are Mean ± SE. * = statistically different.

We first examined the overall change in response time with repeated exposure to the sequence. Figure 2A shows the mean response times for each sequence (S1–S40) across all participants in the young (black) and old (gray) groups. As can be seen in this figure, in general, response times decreased in both groups. However, response times were clearly greater in the older participants and continued to be so even at the end of the learning phase, consistent with aging-related decrease in motor response times. Mean response time on the first presentation of the sequence was 402 ± 14 ms in the young group and 655 ± 65 ms in the older group. These times decreased to 316 ± 19 ms and 511 ± 61 ms in the young and older groups respectively at the end of the sequence block. Introduction of the random trials following 40 presentations of the sequence led to a clear increase in response time in both groups but performance was restored when the sequence was reintroduced (S41, S42), suggesting that participants had acquired knowledge about the sequence. Our group (young, old) × sequence (1 through 40) ANOVA showed a clear effect of group (*F*_(1,24)_ = 12.5314, *p* = 0.0017), with *post hoc* tests confirming larger response times in the older participants compared to the young. The main effect of sequence number was also significant (*F*_(39,936)_ = 10.1826, *p* < 0.0001), pointing to the reduction in response times with learning. However, we did not observe a significant interaction between group and sequence number (*F*_(39,936)_ = 0.7576, *p* = 0.8597) when all 40 sequence repetitions were considered, suggesting that the overall pattern of change in response times was similar across the groups. We also considered the performance of each hand individually and found similar trends. There was a clear reduction in response time of each hand from S1–S40, with no differences between the hands in either group. Both hands showed an increase in response time when the random sequence was introduced, as well as a reduction of response time when the sequence was reintroduced (S41, S42).

### Sequence Specific Learning

Examining the overall pattern of reduction in response times does not elucidate whether sequence specific learning occurred or other generic factors such as comfort with the task led to that reduction. To ensure that sequence-specific learning occurred, and then investigate whether the young and old participants demonstrated differences in sequence-specific learning, we considered multiple approaches. Our first approach relied on absolute time measures; we reasoned that if factors such task comfort impacted task performance, their effects should be evident in both sequential and random trials. Thus, we would see response time decreasing not only from the first to the last sequence presentation, but also between the first and second set of random trials (separated by 40 sequence trials). However, this was not the case. When we compared the magnitude of learning using absolute time measures by assessing the net change in response time from the first to the last sequence repetition (Figure 2B), we found a significant group (young, old) × sequence (first, last) interaction (*F*_(1,24)_ = 5.9470, *p* = 0.0225). We also noted significant group (*F*_(1,24)_ = 13.8134, *p* = 0.0011) and sequence effects (*F*_(1,24)_ = 94.6247, *p* < 0.0001). Follow-up tests showed that while both groups demonstrated a significant change in response times when performing the last sequence compared to the first, the change was greater in the older group (144 ms for the older group vs. 86 ms for the young group). Crucially, such a decrease in response times was not observed between the first and second presentation of the random trials for the young (*p* = 0.6142) or the old (*p* = 0.1320) participants despite a significant interaction effect (*F*_(1,24)_ = 6.2808, *p* = 0.0194), suggesting that the decrease in response times seen during the sequence block cannot be attributed to generic factors such as comfort with the task. Thus, in terms of absolute measures, our older participants demonstrated a greater change in response times over the duration of the sequence practice. Note however that when we accounted for the fact that older subjects were generally slower to respond than the younger participants, and normalized the reduction in response times to the baseline performance (response times in S1), we did not find a significant difference between the groups (*F*_(1,24)_ = 0.1002, *p* = 0.7543). The old group demonstrated a reduction of ~23% while the young group showed a reduction of ~21% in response time over the course of learning, suggesting that in this task, a learning deficit was not evident in the older participants.

Another commonly used measure of sequence specific learning is the difference between response times of the random and sequence trials, often described as a measure of “skill”. Using this measure (difference between second random presentation R2 last and sequence presentation S40), we again noted intact learning in the older participants (Figure 2C). Our ANOVA in fact revealed significantly greater skill in the older group relative to the young (*p* = 0.0359; old mean = 239 ± 28, young mean = 155 ± 26). This finding was consistent with that noted using absolute time measures. However, to also account for the possibility that older participants demonstrated greater learning because they had more room to improve due to longer baseline response times, we normalized the learning measure derived above to baseline performance by dividing the learning measure by the mean response time of the first sequence (S1). Here our ANOVA showed no significant group differences in this normalized learning measure (*p* = 0.2963), indicating again that no learning deficit was evident in older participants relative to the young participants even when their skill level was normalized to baseline performance. In summary, our older participants demonstrated at least as much learning as our younger participants in this bimanual sequential learning task.

### Cost Associated with Switching Hands during Sequence Performance

**Figure 3:**
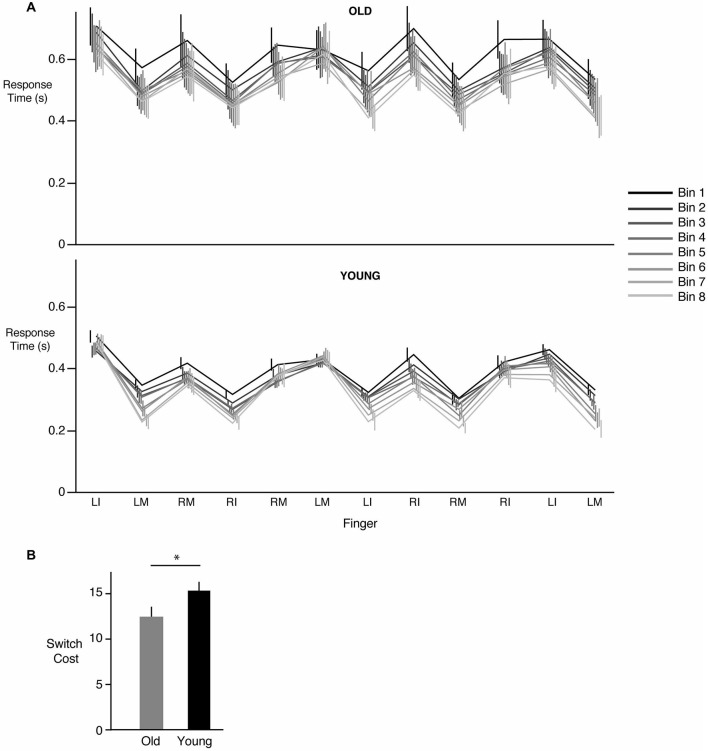
**Changes in response times associated with a hand switch. (A)** Mean ± SE response times for each of the 12 key presses in the training sequence for the old (top panel) and young groups (bottom panel). Data are shown across bins, where each bin represents the mean of 5 sequences. Bin 1 (first 5 sequence presentations) is shown in black, while bin 8 (last 5 sequence presentations) is shown in the lightest shade of gray. Note that the vertical (error bar) lines are slightly offset from each other for better visibility. There was an increase in response time (“switch cost”) whenever a subsequent response required a change in hand (either left to right, or right to left), and this increase remained roughly constant throughout learning. However, a decrease in overall response time from bin 1 to bin 8 was seen. **(B)** Mean ± SE hand switch cost for the old (gray) and the young (black) groups. * = statistically different.

Figure 3A demonstrates the response times for each key press of the 12-element sequence averaged over 5 sequences (a “bin”). The eight bins corresponding to the 40 sequences are shown in different colors, with the black line representing bin 1 (first 5 sequences) and the lightest gray representing bin 8 (last 5 sequences). As can be seen from Figure 2A, response times tended to largely decrease when the same hand was being used to respond to the next stimulus. Interestingly, if the next key press also required use of the same hand (i.e., three consecutive key presses with the same hand), response times either increased or remained the same as before. The reason for such a pattern is unclear (see section Discussion). More importantly however, we consistently observed an increase in response time whenever a switch occurred in the hand being used to make the response. This increased cost associated with making the switch did not change with learning. As can be seen in Figure 3A, the increase in time associated with a hand switch was similar across all bins regardless of group. Thus, in both the young and the older participants, we did not find evidence that learning involved a reduction in hand switch cost. There was however a clear overall decrease in response time from bin 1 to bin 8 in both the young (*p* < 0.0001) and the old groups (*p* < 0.0001). The lack of reduction in switch cost was confirmed statistically; our group (young, old) × bin (1 through 8) ANOVA did not reveal any effect of bin (*F*_(7,168)_ = 0.7385, *p* = 0.6396) or a significant interaction (*F*_(7,168)_ = 0.2551, *p* = 0.9700). However, a main effect of group was observed (*F*_(1,24)_ = 4.19, *p* = 0.04), with *post hoc* tests indicating that switch costs were lower in the older group compared to the young group. This result, illustrated in Figure 3B, indicated that the increase in time associated with a hand switch was smaller in the older compared to the younger participants.

We also considered an alternate analysis where instead of just the trial preceding the switch, we included all trials within a sequence that did not require a switch between hands and used them in the calculation of switch cost. Further, instead of dividing the sequence trials into bins, we examined changes in switch cost, if any, on a sequence-by-sequence basis. This analysis revealed a similar pattern of results in that switch cost for the older participants was smaller than the young participants. Our ANOVA showed neither a significant group × sequence interaction (*F*_(39,936)_ = 0.5737, *p* = 0.9838), nor a significant effect of just sequence number alone (*F*_(39,936)_ = 0.7484, *p* = 0.8702). Instead, as before, we observed a significant group effect (*F*_(1,24)_ = 6.6980, *p* = 0.0098), with substantially smaller switch cost in the older group relative to the younger participants. This indicated that switch cost, while lower in the old group, did not decrease in either group with learning.

### Error Rates

We also compared the accuracy of the key press responses between the young and old subjects using an error rate measure. Error rate was defined as the percentage of trials in which the wrong key was pressed out of the total number of responses on the sequence and random trials. In a two-way ANOVA with group (young, old) and trial type (sequence, random), we found neither a main effect of group (*F*_(1,25)_ = 0.0729, *p* = 0.7893), nor a significant group × trial type interaction (*F*_(1,25)_ = 0.0648, *p* = 0.8011), suggesting that both groups showed a similar pattern of errors for the random and sequential trials. However, there was a main effect of trial type and *post hoc* tests showed that a larger number of errors occurred in the random vs. sequence trials (3.85% vs. 1.27%; *F*_(1,25)_ = 9.5021, *p* = 0.0049). Thus, the larger decrease in response time in the older group as well as the faster switch between hands during response execution was not associated with greater errors in task performance compared to the young group. We also examined whether error rates decreased during the learning of the sequence. However, we did not find a main effect of sequence (*F*_(39,936)_ = 0.9683, *p* = 0.5269), group (*F*_(1,24)_ = 3.1812, *p* = 0.09) or group × sequence interaction (*F*_(39,936)_ = 1.3271, *p* = 0.09), suggesting no particular trend in error rate with learning of the sequence in either group.

## Discussion

We investigated bimanual sequence learning in healthy young and elderly individuals and made a number of key observations: first, both groups of subjects demonstrated intact learning, consistent with several prior reports (Howard and Howard, 1992; Daselaar et al., 2003; Shea et al., 2006; Spencer et al., 2007; Brown et al., 2009; Romano et al., 2010). Second, older participants demonstrated at least as much learning as younger subjects. Third, learning in both groups occurred by means of an overall reduction in response time; we did not observe a reduction in the time taken to switch between hands as learning occurred in either group. However, the overall time cost associated with switching hands when making responses was lower in the elderly group compared to the young subjects.

Our finding that bimanual sequence learning was intact in older individuals is consistent with several studies that have found that aging does not tend to disrupt the capacity to learn procedural skills (Buch et al., 2003; Smith et al., 2005; Heuer and Hegele, 2008; Brown et al., 2009; Anguera et al., 2011; Wang et al., 2011). This has been demonstrated across a range of procedural learning paradigms including motor adaptation, skill learning, as well as sequence learning. Further, we noted equivalent amount of sequence learning in young and old subjects. This is an interesting result since aging is thought to result in a general decline in function across a range of cognitive and motor tasks. For example, it is well known that the speed and accuracy of actions decline with age (Krampe, 2002), and we also noted a decrease in response times in the older group in the current study. In terms of learning however, one study recently demonstrated greater magnitude of learning in an older group relative to young participants in a unimanual SRT paradigm (Brown et al., 2009). Here, while we did not see greater learning in older participants when we normalized the response times to baseline measures, in absolute terms, they did show greater reduction in response time during the course of learning. The exact reason for intact procedural learning in older adults remains unclear. However, if we acquiesce that procedural memory systems interact and compete with declarative memory systems (Brown et al., 2009; Keisler and Shadmehr, 2010; Kantak et al., 2012), and that declarative memory declines with age (Grady et al., 1995; Albert, 1997; Fjell and Walhovd, 2010) along with a reduction in activity of neural structures that support it, an advantage may emerge for the procedural system in normal aging. Thus, the reduction in competition from the declarative system may underlie the maintenance of procedural skills, such as those seen in the current study.

We also assessed whether learning would involve a decrease in the time taken to switch between hands when making key press responses. It is important to recognize that the issue of whether response times increase when a switch of hands is required, itself has been a topic of debate. While some studies reported that inter-response times were shorter when hand switches were required and longer when responses involved different fingers of the same hand (Rabbitt, 1968; Miller, 1982), some other studies have found the opposite (Fox and Stansfield, 1964; Salthouse, 1986). Interestingly, our results about the timing of successive key presses were mixed. While we noted a clear increase in response time when a hand switch occurred, we also found a larger response time for some key presses within a hand. This was particularly evident when three successive responses were of the same hand. In this case, response times showed a clear decrease from the first key press to the second, but tended to increase when the same finger as the first response had to be used for the third response (e.g., RI—RM—RI; Figure 3A). Why this pattern emerges is unclear, but it is possible that subjects expect a switch after two consecutive responses with the same hand, and in the absence of a switch, have to reformulate their response, which takes longer. However, if this is the case, we would expect this effect to diminish as subjects learn the sequence, but this did not occur. At best, subjects maintained the same response time as the previous response, but never showed a decrease in response time on this third key press. Also, it is not clear whether such an effect is characteristic of the sequence being used. For instance, would other sequences that do not involve utilization of the same finger as was recently used, also show a similar trend in terms of response times? Clearly, more work is needed to better understand the mechanism underlying the increase in response time for fingers of the same hand when they are used as in the current study.

Our more consistent finding was that response times increased whenever a switch in hands was required. Our interest was in determining whether this cost associated with hand switching would decrease with learning, and whether this change would be different in the young and older groups. We found no evidence supporting a decrease in switch cost in either the young or the older group. This is in contrast to recent results by Trapp et al. (2012), who showed a decrease in the time taken to switch to a different hand with learning in young, healthy subjects. In our study, regardless of whether the switch occurred from the left to right hand or* vice versa*, the switch cost remained the same throughout the learning phase, and learning was achieved via a global decrease in response times. It is possible that response times and switch costs depend on the probability of occurrence of a switch; response times increase when the probability of occurrence of an event in the sequence is low and this gets reflected as a large switch cost. However, in our case, execution of 4 out of the 12 key presses required a hand switch, which was greater than the probability of a hand switch in the study by Trapp and colleagues (4 out of 15 key presses involved a switch in their study). Yet, we did not observe a decrease in switch cost, arguing against this rationale. Other factors including methodological differences such as use of a different sequence, shorter between-sequence intervals, the lack of explicit awareness of a sequence being presented or the entire sequence not being displayed on the screen all the time in our study may have contributed to the absence of switch cost reduction. Alternatively, if we examine the task from the perspective of a tree-traversal model (Rosenbaum et al., 1983), then then the requirement of traversing an additional node when switching hands is never removed and contributes to the larger switch cost throughout the learning phase.

Interestingly, we observed that overall switch cost was smaller in the older group. This observation indicates that older participants were faster in recruiting the other hand for the task following a response with one hand. Translating this to recruitment of premotor and motor cortical areas, the smaller switch cost implies that the substrates critical for the execution of movements with the other hand were recruited earlier in the older group. Though counterintuitive, this faster recruitment can be explained on the basis of two related lines of work. First, recent work has shown that interhemispheric inhibition, the suppression of corticospinal activity in the contralateral hemisphere to prevent early release of an action, is reduced in older adults compared to young individuals (Talelli et al., 2008; Fujiyama et al., 2009; Hinder et al., 2012; Davidson and Tremblay, 2013; Petitjean and Ko, 2013; Coppi et al., 2014). For instance, Hinder and colleagues showed that inhibition between premotor cortex of one hemisphere and contralateral motor cortex was reduced in older but not younger participants; such reduction could facilitate faster responses in older individuals. Similar results have been previouly reported by Talelli et al. (2008), who demonstrated reduced long-latency inhibition between primary motor cortices in older adults, extending the findings of studies demonstrating reduction in *intra*cortical inhibition with age (Peinemann et al., 2001; Hortobágyi et al., 2006). A second reason for smaller switch costs in older adults could be the often noted bilateral recruitment of motor cortical regions during motor planning and execution. Such bilateral recruitment and reduction in lateralized control has been noted in a number of functional neuroimaging studies using a variety of paradigms (Hutchinson et al., 2002; Ward and Frackowiak, 2003; Naccarato et al., 2006; Rowe et al., 2006; Inuggi et al., 2011; Grady, 2012; Graziadio et al., 2015). This symmetric recrutiment may be the consequence of reduced interhemispheric inhibition or may be a separate mechanism altogether. Nonetheless, if motor control substrates are bilaterally active, as has been suggested to be in older individuals, the time taken to switch from one hand to another could be reduced, reflecting as a smaller switch cost.

In conclusion, we have shown that learning of a simple bimanual sequence appears to be intact in older individuals. Such learning does not necessarily entail a reduction in the time cost associated with switching hands during sequence execution but can be achieved by an overall decrease in response time. However, overall switch cost does appear to be smaller in older individuals relative to younger pariticpants, perhaps reflecting reduced inhibiton or more symmetric recruitment of cortical substrates underlying motor planning and execution.

## Conflict of Interest Statement

The authors declare that the research was conducted in the absence of any commercial or financial relationships that could be construed as a potential conflict of interest.
